# Single-cell transcriptomics reveal intrinsic and systemic T cell aging in COVID-19 and HIV

**DOI:** 10.18632/aging.206353

**Published:** 2026-02-08

**Authors:** Alan Tomusiak, Sierra Lore, Morten Scheibye-Knudsen, Eric Verdin

**Affiliations:** 1 Buck Institute for Research on Aging, Novato, CA 94945, USA; 2 Department of Gerontology, University of Southern California, Los Angeles, CA 90089, USA; 3 Department of Cellular and Molecular Medicine, University of Copenhagen, København 1172, Denmark

**Keywords:** aging, transcriptomic clock, aging biomarkers, systemic aging, intrinsic aging

## Abstract

Biomarkers of aging offer insights into how diseases and interventions affect biological systems. However, most current biomarkers are based on bulk cell measurements, making it difficult to distinguish between changes driven by shifts in cell type composition (systemic effects) versus intrinsic changes within individual cells. To address this, we used single-cell RNA sequencing to analyze aging-related changes at both the cellular and bulk levels. We developed *Tictock* (T immune cell transcriptomic clock), a single-cell transcriptomic clock capable of predicting age and cell type across six human T cell subsets. Applying *Tictock*, we found that acute COVID-19 is associated with increased proportions of CD8+ cytotoxic T cells, whereas T cell composition remains stable in people with HIV on antiretroviral therapy (HIV+ART). Both COVID-19 and HIV+ART are linked to an increase in transcriptomic age, specifically within naïve CD8+ T cells. Gene Ontology enrichment of 209 genes shared across six clock models identified common pathways including the cytosolic small ribosomal subunit, TNF receptor binding, and cytosolic ribosome components. A correlation was also observed between aging and mean transcript length. These findings underscore the promise of single-cell transcriptomic biomarkers to disentangle the systemic and cell-intrinsic components of immune aging and to measure immune aging.

## INTRODUCTION

Aging is a complex, systemic process that involves changes in cellular function, cell composition, tissue organization, and intercellular communication networks [1–3]. Developing biomarkers capable of measuring aging on each level of organization is critical for understanding how molecular and physiological changes occur across the human lifespan. Although numerous tools have been designed to quantify aging, most existing biomarkers rely on bulk analysis of cell populations and therefore focus on a single level of organization. This limitation hampers our ability to distinguish the relative roles of cell population changes and cell-intrinsic aging processes. Creating novel interpretable tools that capture this complexity is critical for the development of interventions to improve human healthspan and lifespan.

Early efforts to design accurate aging biomarkers, often referred to as “clocks,” focused on single molecular measurements. The first clocks were identified by Hannum and Horvath and colleagues based on changes in DNA methylation in 2013 [4, 5]. These clocks predicted biological age using CpG markers derived from DNA methylation data. Over time, clocks were developed based on bulk transcriptomics [6], proteomics [7, 8], ATAC-Seq [9], and other molecular measurements. These advancements have led to new methods for understanding aging at different levels of resolution, each providing unique insights into the aging process. However, the interpretation of these biomarkers can be challenging due to their reliance on measurements derived from a bulk collection of cells or plasma.

To solve this challenge, clocks based on single-cell measurements have been developed. In 2021, Trapp et al. developed a clock based on single cell methylation data [10]. In 2022, Buckley et al. used single-cell transcriptomic profiling data to predict chronological and biological age [11]. Using transcriptomics instead of DNA methylation identifies markers that are easier to link to changes in specific proteins and pathways, thereby providing novel insights into changes that occur with biological age [12, 13]. Furthermore, cell-type specific effects of aging and rejuvenation can be measured. As an example, Yu et al. (2023) used a technical innovation on single-cell transcriptomic data to derive novel insights regarding the effect of exercise on neuronal rejuvenation [14]. Similarly, single-cell chromatin measurements based on imaging have recently been developed to quantify age reversal by partial reprogramming [15]. This development enables scientists to explore how individual cells within an organism age differently, contributing to our understanding of cellular heterogeneity in aging.

Concurrently, several groups developed biomarkers that predict aging using unique clinical measurements. As an example, Levine et al. (2018) used methylation data to predict several key healthspan measurements. GrimAge used a similar technique to predict time-to-death, time-to-coronary heart disease, and time-to-cancer [16, 17]. More recently, clocks have been developed to predict the aging of individual organs [18] and physiological systems [19]. These measurements allow for better understanding of systemic changes directly relevant to human health.

Several studies have integrated aging at several levels of resolution simultaneously, frequently by employing multi-omics. In the context of human aging, these measurements have led to improved clinical predictions [20] and the discovery of age-associated chemokines [21]. Other groups have combined multi-omics with longitudinal data, enabling a deeper understanding of the dynamics of human aging [2, 22]. Combining measurements of systemic aging with advancements in single-cell biomarkers has the potential for unlocking a deeper understanding of the interplay between extrinsic and intrinsic aging.

Simultaneous profiling of intrinsic and systemic aging has particular importance in the context of the immune system [23]. Due to both intrinsic (i.e., cell autonomous) and extrinsic factors (e.g. thymic involution), the naïve CD8+ and CD4+ T cell compartments decline over time [24], both in size and in quality. This decline impairs the ability of the organism to mount immune responses during aging and to fight novel infections [25]. Thymic involution leads to changes in cell-type composition via a decline in naïve CD4 and CD8 T cells and a concomitant increase in memory and effector T cells [23, 26–28]. This complex interplay of cellular and systemic aging is important, but our ability to assess how each of these independently contributes to aging-associated pathology is presently limited.

Here, we use a previously published single-cell transcriptomic dataset [23] to generate predictors of T cell composition and individual cellular aging. We demonstrate that our cell type predictor can identify and quantify six canonical T cell subsets (naïve CD8s, central memory CD8s, effector memory CD8s, naïve CD4s, central memory CD4s, and regulatory T cells) and their changes in relative abundance during aging. Using this cell type predictor, we generated six individual age predictors for each predicted T cell subtype. We then applied our joint cell type and age prediction models, collectively known as *Tictock* (T immune cell transcriptomic clock), to two datasets – one of acute COVID and another of HIV-infected individuals with long-term ART treatment. Similar to what we have shown previously using epigenetic data [29], we find that acute COVID is associated with changes in cell type composition. We further show that both diseases are associated with intrinsic accelerated aging in naïve CD8 T cells. Lastly, we investigate the mechanistic drivers of our age predictors and identify associations with ribosomal gene expression and mean cell transcript length.

To provide a unified framework for both systemic and cell-intrinsic immune aging, we integrated two complementary predictive models within *Tictock*. The first model performs automated cell type classification of peripheral T cells into six canonical subsets (naïve CD8, central memory CD8, effector memory CD8, naïve CD4, central memory CD4, and regulatory T cells) using multinomial logistic regression trained on marker gene expression profiles. The second model comprises six independent age-prediction models, each optimized for one of the identified cell types through elastic net regression on age-correlated genes. Together, these models allow joint inference of cell identity and transcriptomic age from any single-cell dataset. This dual-layer design enables us to disentangle compositional (systemic) changes from intrinsic transcriptional aging within immune cell subsets and to apply the integrated model to disease contexts such as COVID-19 and HIV. Because each age model is trained and applied within a single T cell lineage, predicted ages should not be interpreted as global PBMC-level chronological age estimates. Instead, *Tictock* is intended to quantify relative, cell-intrinsic aging states within defined T cell subsets, enabling comparisons across conditions and disease contexts.

## RESULTS

### Automated prediction of cell type recapitulates known changes in T cell composition with age

**Figure 1:**
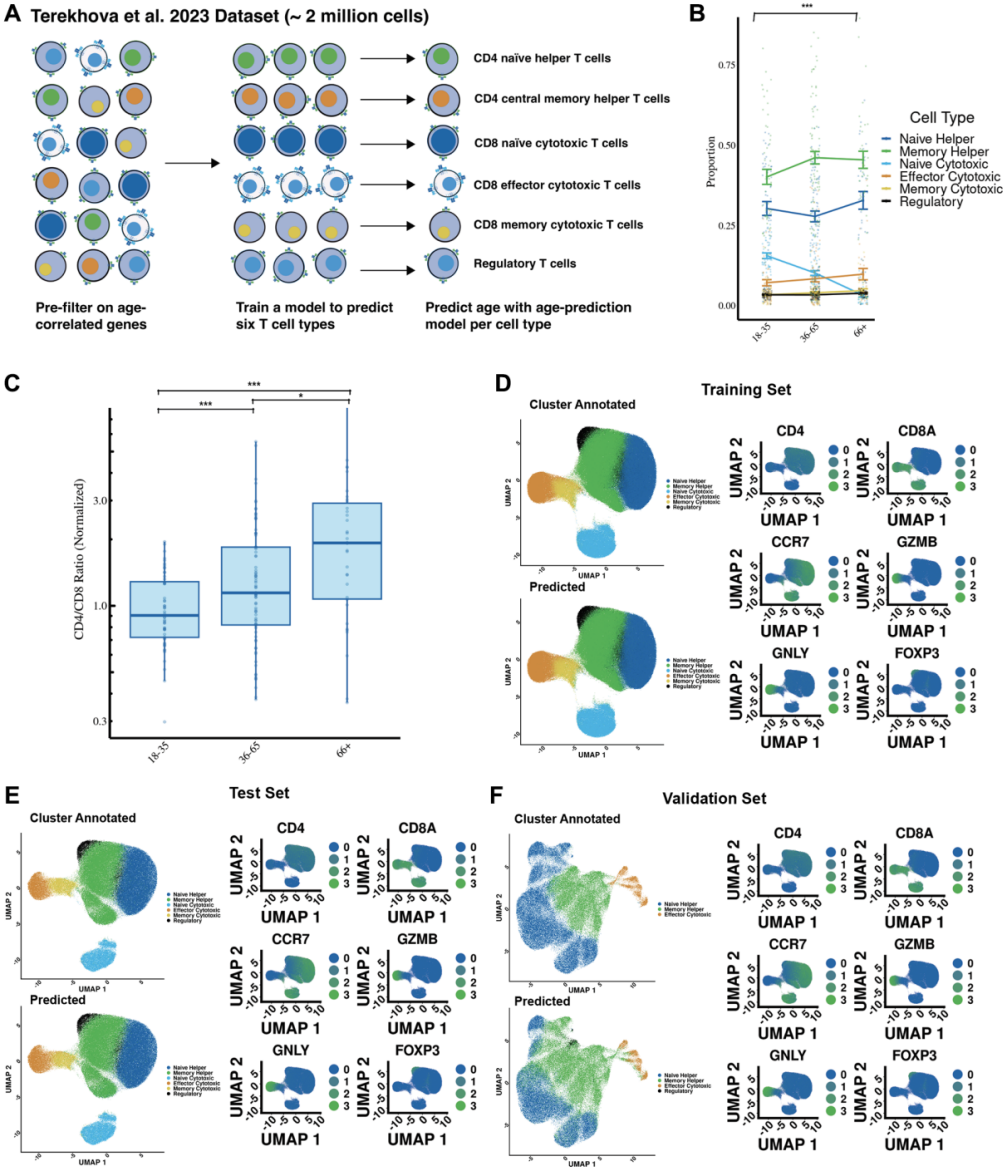
**Cell type predictions recapitulate known effects of aging on the immune system.** (**A**) Overview of the study design, including the development of cell type prediction and age prediction models using single-cell transcriptomics. The workflow highlights data preprocessing, clustering, annotation, and model training. (**B**) Cell type proportion changes in individuals from different age groups. (**C**) CD4/CD8 ratio increases with age, normalized to individuals aged 18–35. (**D**) Comparison of predicted versus manually annotated cell types in the training dataset. Cell annotations were based on canonical markers: CD4, CD8A, CCR7, GZMB, GNLY, and FOXP3. Predicted clusters align closely with ground truth annotations, demonstrating the accuracy of the model. (**E**) Validation of the model in the test dataset, showing high concordance between predicted and manually annotated clusters with quantitative accuracy metrics (97% accuracy; F1 score = 0.97). (**F**) External validation using the Yasumizu et al. (2024) dataset demonstrates robustness across datasets (83% accuracy; F1 score = 0.80). Statistical significance is indicated: ^***^ Bonferroni-corrected *p*-value less than or equal to .001, ^*^ Bonferroni-corrected *P*-value less than or equal to .05, ^#^ Bonferroni-corrected *P*-value less than or equal to .1.

To simultaneously profile changes in blood cell type composition and intrinsic age-associated changes, we have developed separate models to build *Tictock*: a cell type prediction model and six age prediction models for each cell type (naïve CD8s, central memory CD8s, effector memory CD8s, naïve CD4s, central memory CD4s, and regulatory T cells). Automating cell type prediction allows for less bias during cellular age prediction. As the basis for our model, we use a previously published scRNA-Seq dataset of two million peripheral blood mononuclear cells (PBMCs) from 166 individuals [23]. As noise can significantly affect accurate cell type or age prediction, we filter our analysis to include only genes showing at least a weak correlation with age (|R| >0.01). This step reduces technical noise and standardizes the gene set across models while retaining highly expressed canonical T-cell markers such as CD4, CD8A, CCR7, GZMB, GNLY, and FOXP3. However, we acknowledge that this approach may exclude genes whose expression changes non-linearly with age, as reported in recent studies [2] (Figure 1A).

The dataset is next split on a per-donor basis into a training subset (80%) used for building the model and a test subset (20%) for determining its accuracy and precision. We use an additional external dataset [30] to measure the ability of *Tictock* to make accurate predictions in other cohorts. To determine cell prediction accuracy and generate initial labels, we use K-means clustering to split the cells into biologically relevant groups and then label these groups based on the expression levels of six cell surface markers (CD4, CD8A, CCR7, GZMB, GNLY, and FOXP3). Cell subsets are identified based on the following positive markers: naïve CD4 helper T cells (CD4, CCR7), central memory CD4 helper T cells (CD4), CD8 naïve cytotoxic T cells (CD8A, CCR7), CD8 effector cytotoxic T cells (CD8A, GZMB, GNLY), CD8 memory cytotoxic T cells (CD8A), and regulatory T cells (FOXP3, CD4).

We validate our model by first identifying whether previously reported aging-associated trends could be recapitulated. In agreement with previously identified and reported changes [28], we identify an increase in the CD4/CD8 ratio with age (*p* = .0003) (Figure 1B). Within the six cell types we measured, we observe a significant decrease (*p* < .0001) in the proportion of CD8 naïve cytotoxic cells (Figure 1C) with age, which is also in accordance with previous literature [31].

We further test our cell type prediction model by comparing predicted cell types to those we manually annotated. In both the training (Figure 1D) (97% accuracy; .98 F1 score) and test (Figure 1E) (97% accuracy; .97 F1 score) datasets, the predicted cell types closely match the manually annotated clusters. Furthermore, there is accordance with cell types as identified by canonical cell markers CD8A, CD4, CCR7, GZMB, GNLY, and FOXP3. To further assess generalizability, we test our cell type prediction model using an external dataset of CD4+ T cells [30]. We identify strong accordance (83% accuracy; .80 F1 score) between the manual annotation and assumed clusters based on canonical cell markers (Figure 1F).

### Cell type-dependent models predict age across a variety of cell types

After validating our cell type prediction model, we next developed six independent age prediction models—one for each of the six predicted cell types (naïve CD8s, central memory CD8s, effector memory CD8s, naïve CD4s, central memory CD4s, and regulatory T cells). We employ elastic net regression as our modeling approach, which enables us to focus on the most informative features while keeping our models both straightforward and robust. We tune the parameters through cross-validation to ensure that the models could capture the subtle signals of aging. Each model provides a predicted age for individual cells, which we compare to the donors’ chronological ages at both the single-cell level and as an average per donor. This comprehensive approach not only validates our predictions but also enhances our understanding of how these immune cell types mirror the aging process, ultimately linking cellular signatures to overall donor age.

**Figure 2:**
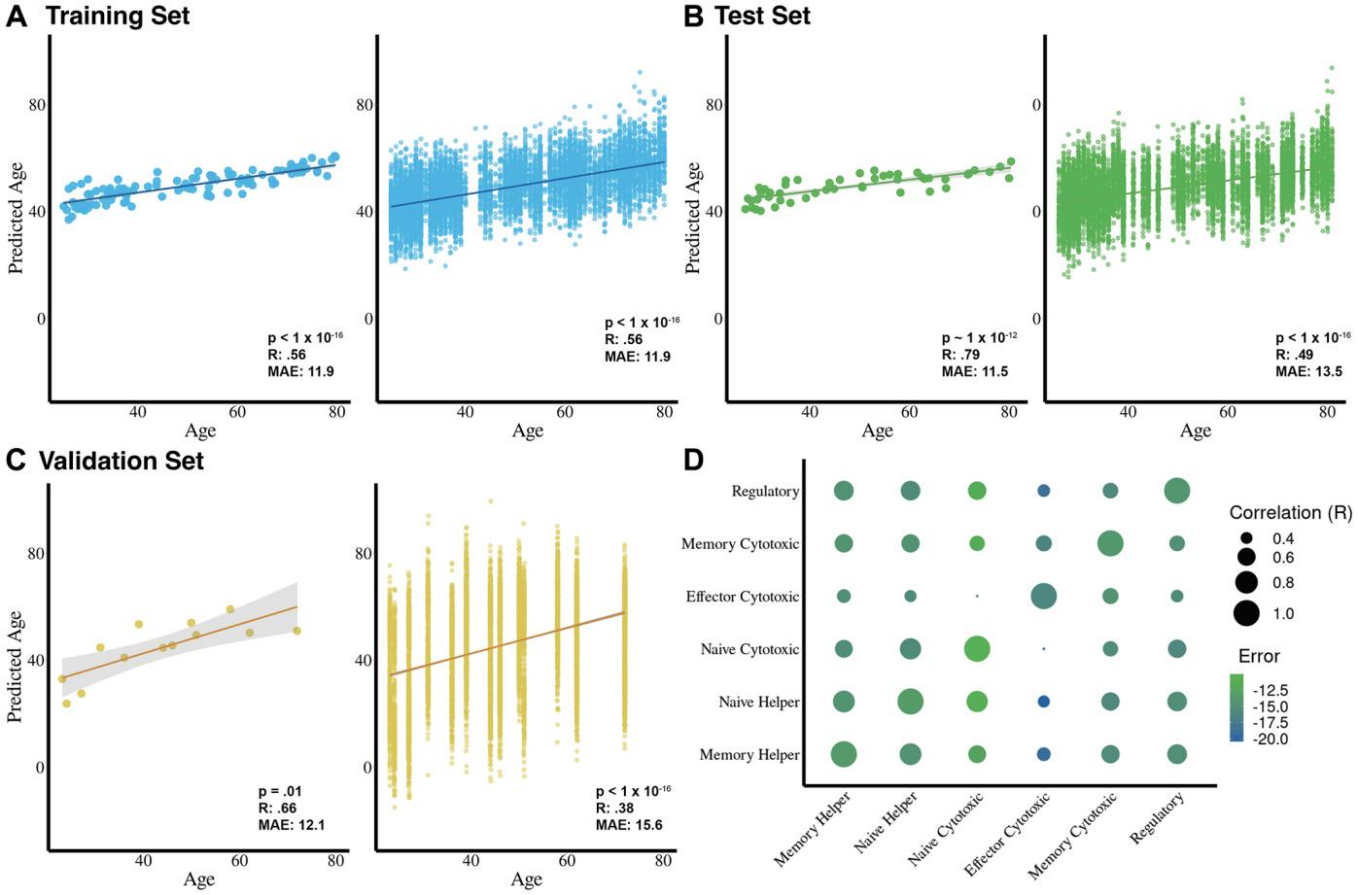
**Cell-type-specific age prediction models predict chronological age with high accuracy and reveal unique aging signatures.** (**A**) Training dataset results (*n* = 116 donors) show strong correlations between predicted and chronological age at the donor level (left; R = 0.84, MAE = 11.4 years, *p* < 1 × 10^−16^) and at the cell level (right; R = 0.56, MAE = 11.9 years, *p* < 1 × 10^−16^). (**B**) Test dataset results (*n* = 50 donors) show strong correlations at the donor level (left; R = 0.79, MAE = 11.5 years, *p* < 1 × 10^−12^) and moderate correlations at the cell level (right; R = 0.49, MAE = 13.5 years, *p* < 1 × 10^−16^). (**C**) External validation using the Yasumizu et al. (2024) dataset (*n* = 13 donors) reveals strong correlations at the donor level (R = 0.78, MAE = 6.5 years, *p* = 0.001) and moderate-to-low correlations at the cell level (R = 0.38, MAE = 15.2 years, *p* < 1 × 10^−16^). (**D**) Pairwise correlations between age predictors for each T cell subset highlight both shared and cell-type-specific aging signatures, with relative errors shown for each subset clock compared to donor chronological age on the test dataset.

For the training set, we observe a strong correlation between the predicted age of a cell and chronological age (R = .56, mean absolute error (MAE) = 11.9, *p* < 1 × 10^−16^), particularly when the average age of all cells per donor is calculated (R = .84, MAE = 11.4, *p* < 1 × 10^−16^) (Figure 2A). As expected for a lineage-restricted aging clock, donor-level averaged predictions occupy a narrower age range than chronological age, reflecting shared transcriptional constraints within each T cell lineage rather than a limitation of model performance. Similarly, we observe a moderate correlation in the test set (R = .49, MAE = 13.5, *p* < 1 × 10^−16^), which also increases when we compute the average age of all cells per donor (R = .8, MAE = 11.5, *p* < 1 × 10^−12^) (Figure 2B). We apply *Tictock* to the Yasumizu et al. (2024) dataset and observe a weaker but still significant correlation on a cell level (R= .38, MAE = 15.2, *p* < 1 × 10^−16^), and when cells are averaged per each donor (R = .78, MAE = 6.5, *p* = .001) (Figure 2C). Because *Tictock* consists of six cell-type–specific clocks trained within individual T cell subsets, the donor-level predicted ages occupy a narrower absolute range than the full chronological span of the cohort. This reflects the restricted transcriptional variation that is available within a single immune lineage and is expected for lineage-specific single-cell models [28, 32]. In this context, the primary output of interest is the relationship between predicted and chronological age, particularly after averaging predictions at the donor level, rather than the absolute magnitude of the predicted values [11]. Consistent with this, age-differential calculations showed donor-level ranges of approximately −25 to +23 years in the Terekhova et al. training cohort, −28 to +19 years in the Terekhova et al. test cohort, −10 to +29 years in the Yasumizu et al. dataset, −21 to +37 years in Ren et al. (COVID-19), and −15 to +21 years in Wang et al. (HIV+ART).

We also note that predictions at the single-cell level exhibit a wide dispersion. This reflects the sparsity and stochastic variability inherent to scRNA-seq data, where each cell captures only a fraction of its true transcriptome [33, 34]. Such dispersion is a well-recognized feature of single-cell predictive modeling. As in other single-cell applications, averaging predictions at the donor level substantially reduces this variability and yields stable and biologically meaningful associations with chronological age.

To determine whether each of these age predictors is identifying unique cell type-specific aging patterns, we test whether they are correlated with each other. In general, the individual age predictors have moderate (R ~ .5) age prediction correlations with one another, suggesting they measure both a shared and cell-type dependent aging signature (Figure 2D).

### COVID-19 impacts immune aging through both cell composition and intrinsic aging mechanisms

Aging and diseases such as COVID-19 have proven to have significant impacts on T cell composition and immune function [35]. To test whether acute COVID-19 impacts cell type composition, cellular aging, or both, we use an external COVID-19 dataset made up of 171 COVID-19 infected and 25 healthy individuals [35], filtering on individuals who had COVID-19 within 60 days of sample collection. To ensure biological interpretability, we restricted the COVID-19 cohort to donors sampled within 60 days of symptom onset, which captures acute and early convalescent immune perturbations while avoiding later post-infectious time points that show substantial heterogeneity in immune recovery.

**Figure 3:**
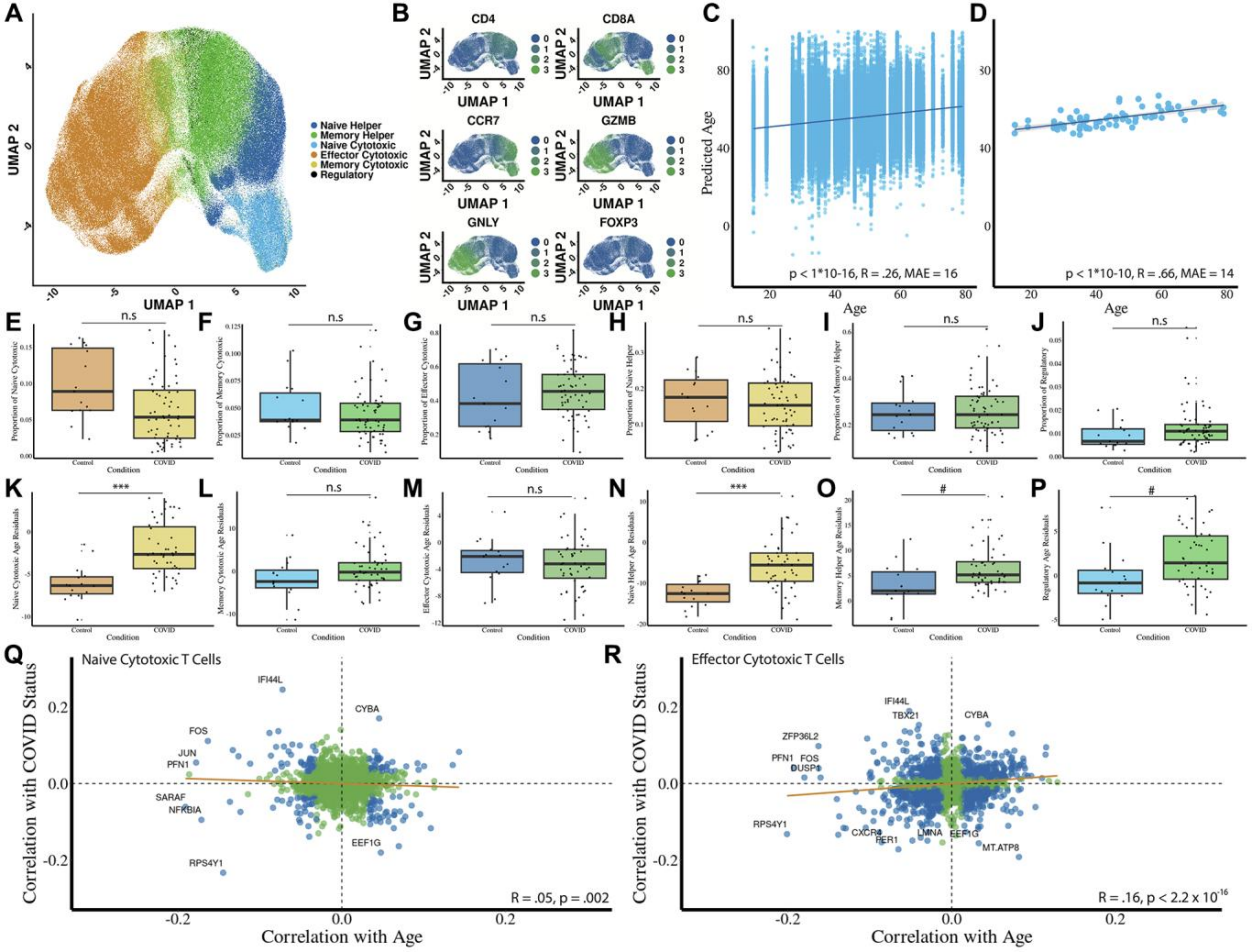
**Acute COVID-19 impacts T cell composition, cellular aging, and gene expression through both systemic and intrinsic mechanisms.** (**A**) Predicted cell types using the cell type prediction model (*n* = 15 controls; *n* = 48 donors with COVID-19). (**B**) Heatmap of canonical marker expression across predicted subsets validates the cell type predictions. Markers include CD8A, CD4, CCR7, GZMB, GNLY, and FOXP3. (**C**) Predicted age of individual T cells versus chronological age of donors shows a low correlation (R = 0.26, MAE = 16 years, *p* < 1 × 10^−16^). (**D**) Averaged predicted donor ages versus chronological age shows a moderate-to-strong correlation (R = 0.66, MAE = 14 years, *p* < 1 × 10^−10^). (**E**–**J**) Proportional changes in each T cell subset due to COVID-19. (**K**–**P**) Residual age prediction by condition for each cell type due to COVID-19. (**Q**, **R**) Gene-level analysis of shared transcriptional signatures between COVID-19 and aging in naïve and effector CD8 T cells. Correlations are shown with regression lines (orange) and statistical significance marked in blue. Statistical significance is indicated: ^***^ Bonferroni-corrected *p*-value less than or equal to .001, ^*^ Bonferroni-corrected *P*-value less than or equal to .05, ^#^ Bonferroni-corrected *P*-value less than or equal to .1.

Applying our cell type prediction model to this dataset (Figure 3A), we find identification of distinct T cell subsets to be in accordance with cell markers (Figure 3B). Next, using the age prediction models, we evaluate the predicted ages of individual cells and the predicted ages of donors. We find that *Tictock* achieved moderate accuracy on individual cells (R = .26, MAE = 16, *p* < 1 × 10^−16^) (Figure 3C) and cell ages averaged per donor (R = 0.66, MAE = 14, *p* < 1 × 10^−10^) (Figure 3D).

To determine whether acute COVID-19 infection affects cell type composition, we analyze predicted proportions across T-cell subsets. We observe significant decreases in predicted proportions of naïve CD8 (Figure 3E; *p* = .03) and naïve CD4 cells (Figure 3H; *p* =.03). We observe weaker changes in cell type proportions for other cell types, including CD8 central memory cells (Figure 3F, *p* = .21), CD8 effector cells (Figure 3G, *p* = .3), CD4 central memory cells (Figure 3I, *p* = .83), and regulatory T cells (Figure 3J, *p* = .08). As naïve cells make up approximately 30% of cells in the patient samples, lowering their relative proportion is a significant immunological impact.

Next, we test whether predicted cellular ages are affected by COVID-19. Because COVID-19 donors were older than controls (Supplementary Figure 1), all disease-associated effects were evaluated using age residuals, which remove chronological age from the predicted transcriptomic age. We observe significant age acceleration in naïve CD8 (Figure 3K, *p* = 1e^−6^) and CD4 memory (Figure 3O, *p* = .02) cells. Naïve CD8 T cells showed substantial age acceleration in COVID-19 (mean age residual difference ≈ 3.6 years, 95% CI (2.1, 5.1), Cohen’s d ≈ 1.15), while memory CD4 T cells displayed a more moderate shift (mean difference ≈ 2.8 years, 95% CI (0.17, 5.41), Cohen’s d ≈ 0.63). In contrast, naïve CD4 helper cells do not show evidence of intrinsic age acceleration (Figure 3N, *p* = 1e^−7^) as their mean residual is slightly negative in COVID-19 donors. We did not see a significant shift in predicted age for CD8 memory (Figure 3L, *p* = .2), CD8 effector (Figure 3M, *p* = .98), or regulatory (Figure 3P, *p* = .07) T cells. Finally, we examine whether aging and COVID-19 share a transcriptional signature between aging and COVID-19 in naïve CD8 and effector CD8 cells. There is only a slight negative (Figure 3Q, R = −.05, *p* = .002) and a slight positive (Figure 3R, R = .16, *p* < 1 × 10^−16^) correlation between COVID-19 and aging in naïve CD8 and effector CD8 cells, respectively.

### Long-lasting ART treatment and HIV impacts aging through cell-intrinsic mechanisms

HIV has been previously reported to accelerate aging [36–38]. We were interested in testing whether this accelerated aging is due to cell-intrinsic or systemic effects. After validation of *Tictock* on the Ren et al. (2021) COVID-19 dataset and exploring the effects of T cell changes with age and disease, we used another external dataset comprised of patients infected with human immunodeficiency virus (HIV) on antiretroviral therapy and healthy controls [39]. Of these donors, 7 had HIV and were on antiretroviral therapy (ART), 1 had HIV but was off ART for the first visit, and 6 donors were healthy.

**Figure 4:**
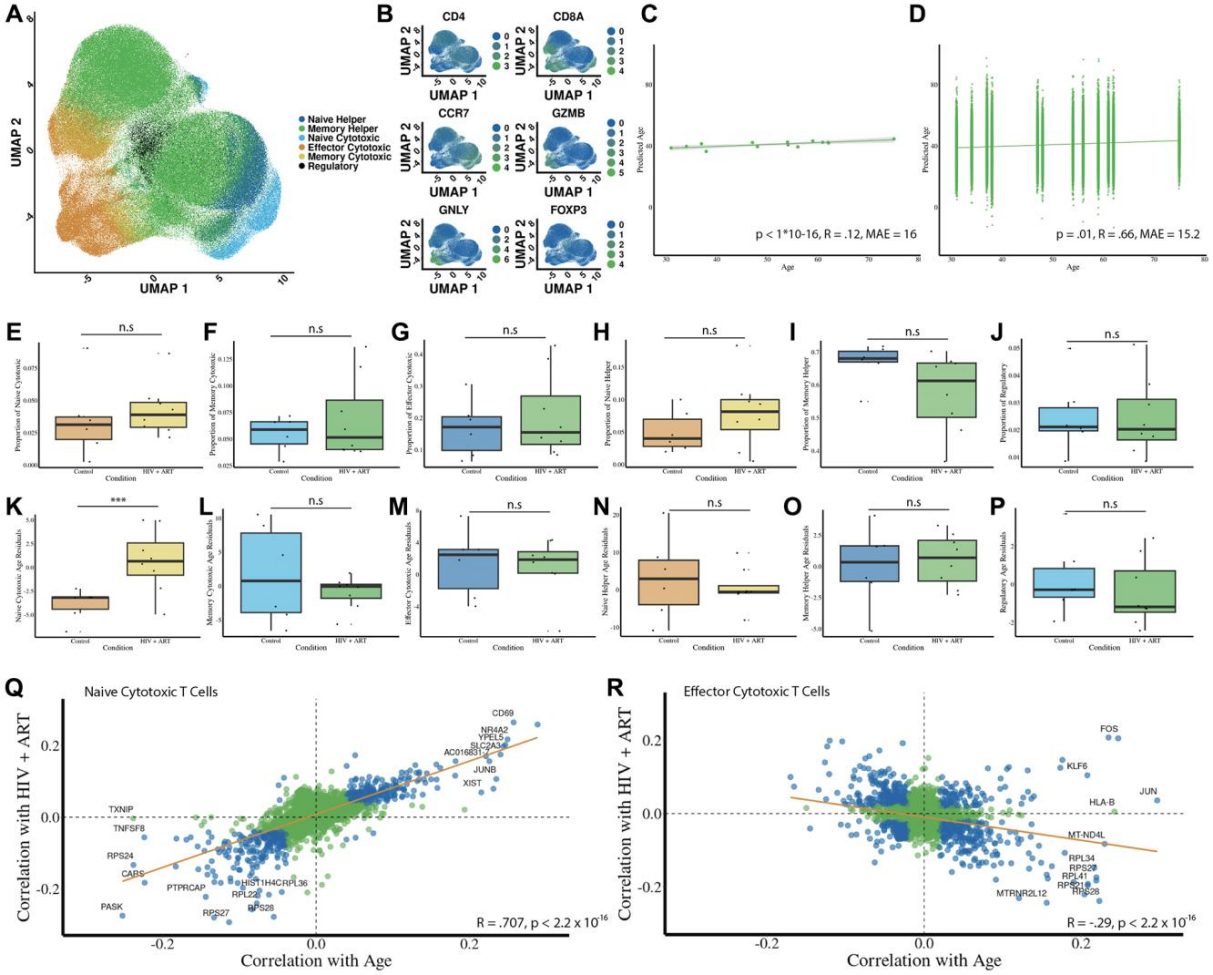
**HIV+ART accelerates intrinsic aging in naïve CD8 T cells while maintaining stable cell type proportions.** (**A**) Predicted cell types using the cell type prediction model (*n* = 6 controls; *n* = 8 donors with HIV). (**B**) Heatmap of canonical marker expression across predicted subsets validates the cell type predictions. Markers include CD8A, CD4, CCR7, GZMB, GNLY, and FOXP3. (**C**) Predicted age of individual T cells versus chronological age of donors shows a low correlation (R = 0.14, MAE = 13.6 years, *p* < 1 × 10^−16^). (**D**) Averaged predicted donor ages versus chronological age shows a strong correlation (R = 0.66, MAE = 12.6 years, *p* =.002). (**E**–**J**) Proportional changes in each T cell subset due to HIV + ART. (**K**–**P**) Residual age prediction by condition for each cell type due to HIV + ART. (**Q**, **R**) Gene-level analysis of shared transcriptional signatures between HIV + ART and aging in naïve and effector CD8 T cells. Correlations are shown with regression lines (orange) and statistical significance marked in blue. Statistical significance is indicated: ^***^ Bonferroni-corrected *p*-value less than or equal to .001, ^*^ Bonferroni-corrected *P*-value less than or equal to .05, ^#^ Bonferroni-corrected *P*-value less than or equal to .1.

We apply our cell type prediction model to this dataset, successfully identifying and clustering the six distinct T cell types (Figure 4A) and finding the clusters to be in accordance with known cell markers (Figure 4B). Next, we use the age prediction models on this HIV dataset to predict the age of individual cells (Figure 4C) and donors (Figure 4D). We observe a low accuracy for age prediction comparing predicted cell age to donor age (R = .14, MAE = 13.6, *p* < 1 × 10^−16^) but a strong accuracy when cell age predictions are averaged per donor and compared to chronological age (R = 0.66, MAE = 12.6, *p* = .002). Age differential calculations showed that donor-level predicted ages are compressed relative to chronological age. Therefore, all disease comparisons use age residuals to correct this bias. Unlike the COVID-19 dataset, we do not observe significant changes in cell type proportions after adjusting for multiple comparisons (Figure 4E–4J). To assess differences in predicted age between HIV + ART donors compared to healthy controls, we perform an analysis of the age residuals for each cell type. We identify significantly accelerated ages in HIV+ patients in the naïve CD8 population (Figure 4K, *p* = .01), while all other T cell subsets show no significant age changes (Figure 4L–4P). In HIV+ART donors, naïve CD8 T cells also exhibited increased transcriptomic age (mean age residual difference ≈ 5.4 years, 95% CI (0.95, 9.85), Cohen’s d ≈ 1.48).

Similarly to the COVID dataset, we examine transcriptional signatures of HIV+ART relative to aging. In naïve CD8 cells (Figure 4Q), we observe a strikingly strong correspondence between genes that have a partial correlation with aging and those that have a partial correlation with HIV+ART (R = .7, *p* < 1 × 10^−16^). Interestingly, many of the genes most strongly correlated with both HIV+ART and aging have been previously characterized to be a signature of HIV, such as CD69 [40]. We also find a weaker negative correlation between genes associated with aging and HIV in CD8 effector cells (Figure 4R, R = −.29, *p* < 1 × 10^−16^).

### Biological insights into aging using the six T cell type-dependent model coefficients

One of the distinct advantages of using scRNA-Seq data or transcriptomics in comparison to other measurements of aging is the proximity to readily identifiable changes in biological function. In other clocks, such as those built using DNA methylation data, the interpretability of the clocks to gain information about the biology of aging is often limited [41].

**Figure 5:**
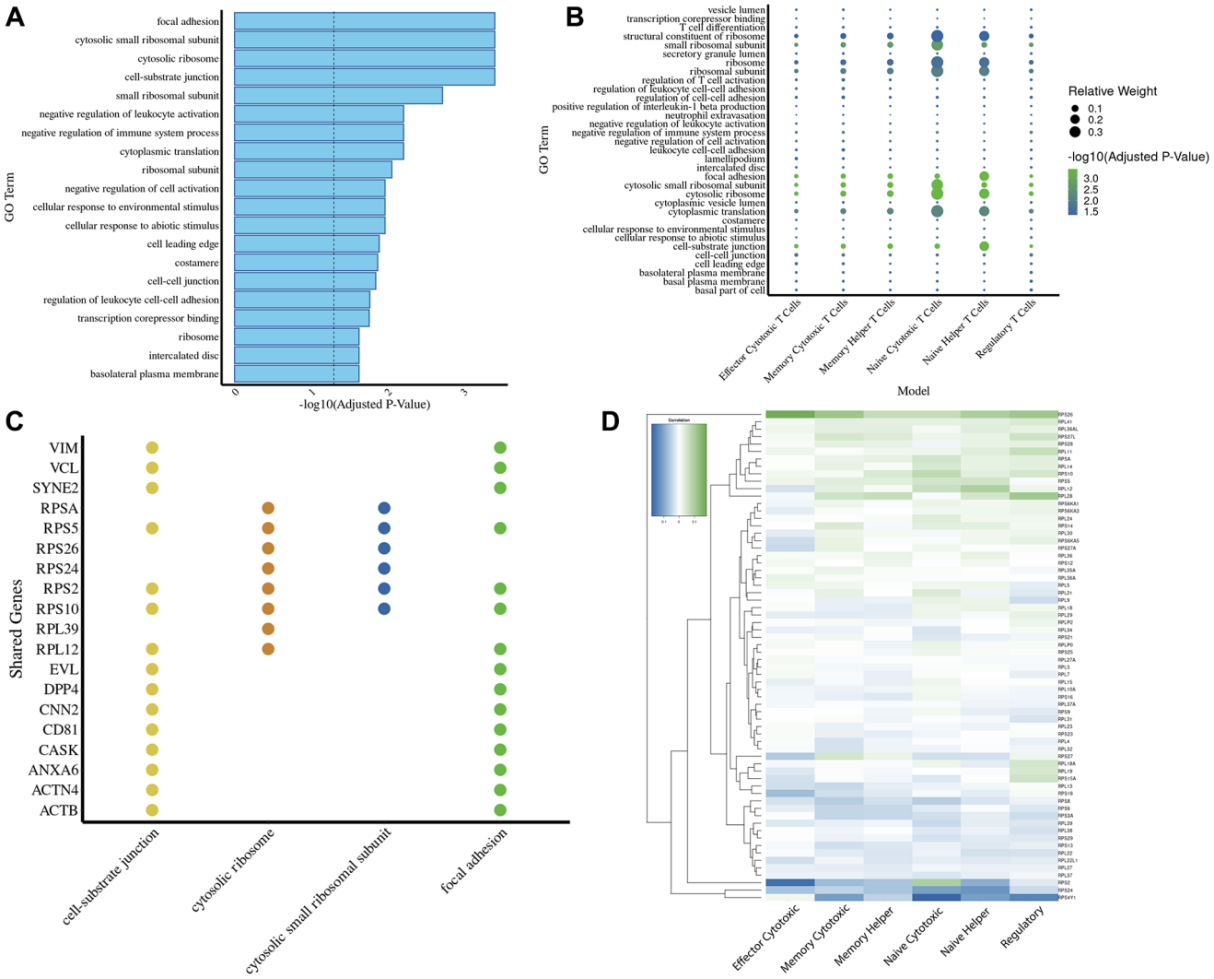
**Gene ontology and chromosomal mapping highlight ribosomal pathways as key drivers of transcriptomic aging predictions.** (**A**) GO term enrichment analysis of genes shared across all six T cell age prediction models reveals significant enrichment in ribosomal pathways, cell-substrate junction, focal adhesion, and cytosolic processes. The dashed line marks the significance threshold (-log10(p) = 1.3). (**B**) A dotplot for the GO enrichment analysis of the genes used in every model (using the relative weights of those genes in relation to the rest of the genes used in the model). (**C**) A bar plot displaying the top four most enriched GO terms ranked by adjusted *p*-value. For each GO term, the top four contributing genes were identified and compared across all enrichment terms, highlighting shared and unique functional associations among the gene sets. (**D**) Heatmap showing correlations of individual ribosomal gene expression with aging across T cell subsets, highlighting conserved and subset-specific trends.

We focus on the subset of genes used by each age prediction model to conduct a broad exploratory analysis (Supplementary Figure 2). Accordingly, we perform a GO enrichment of the 209 shared genes across all six T cell age prediction models, revealing significant enrichment in ribosomal pathways, cell-substrate junction, focal adhesion, and cytosolic processes (Figure 5A). Mapping these shared predictive genes to their genomic locations revealed non-random clustering, with particularly dense regions on chromosomes 1 and 14 (Supplementary Figure 3). Next, we perform a deeper GO enrichment analysis of the individual T cell models. This is based on the total gene set specific to that individual model, weighting genes by their relative contribution (Figure 5B). This allows us to determine the functional significance of genes within each model (Supplementary Figure 4), highlighting that both naïve cytotoxic T cells and naïve helper T cells show particularly high involvement of ribosomal pathways.

Building on our earlier analyses, we delve deeper into the functional associations underlying our enriched GO terms. We present a bar plot that ranks the top four GO terms by adjusted *p*-values and highlights their top four contributing genes, revealing both shared and distinct functional signatures among the gene sets (Figure 5C). Recognizing the critical role of ribosomal genes, we further examine their expression dynamics with age across different cell types (Figure 5D and Supplementary Figure 5). While most ribosomal genes exhibit a consistent age-associated pattern across all six cell types, exceptions like RPS2 suggest the presence of unique regulatory mechanisms.

**Figure 6:**
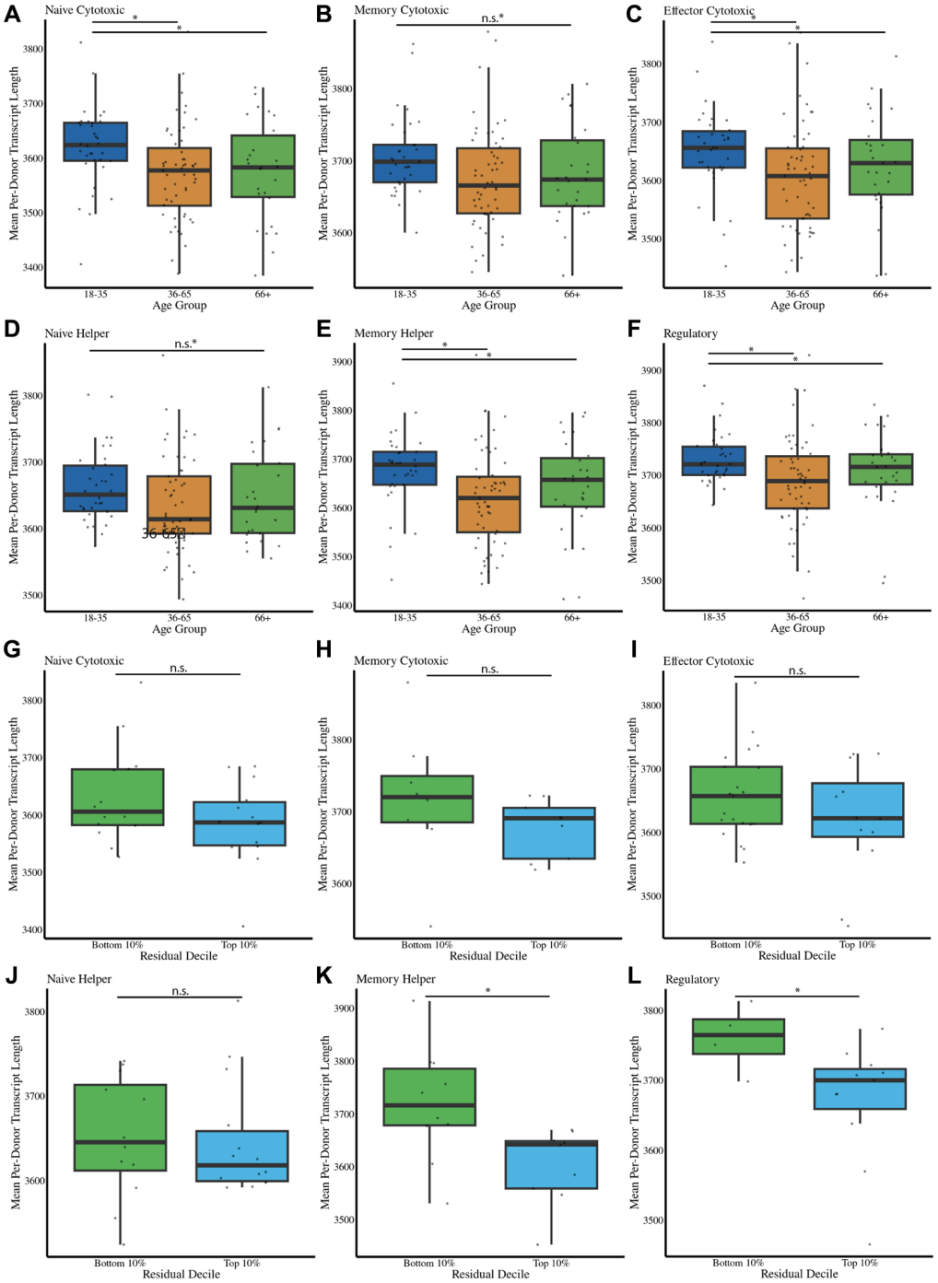
**Transcript length is associated with cellular aging and predicts accelerated aging in specific T cell subsets.** (**A**–**F**) Mean transcript length per cell type and age group for naïve CD8, memory CD8, effector CD8, naïve CD4, memory CD4, and regulatory T cells. Shorter transcript lengths are associated with increased age for most subsets, except naïve CD4 and memory CD8 T cells. (**G**–**L**) Transcript length comparisons in cells from the top and bottom 10% deciles of predicted age acceleration relative to chronological age. Longer mean transcript lengths are associated with younger predicted ages in regulatory T cells and memory CD4 cells. Statistical significance is indicated: ^***^ Bonferroni-corrected *p*-value less than or equal to .001, ^*^ Bonferroni-corrected *P*-value less than or equal to .05, ^#^ Bonferroni-corrected *P*-value less than or equal to .1.

Due to increasing evidence that defective transcription of longer transcripts is associated with aging [42, 43], we sought to identify whether such defects could be identified in the immune system during human aging using our single-cell transcriptional biomarker. We find that older individuals had, on average, shorter transcript lengths in every T cell subset except for naïve CD4 T cells and memory CD8 T cells (Figure 6A–6F). To test whether shorter transcript lengths are associated with an accelerated predicted age, we estimate transcript lengths in cells in the top and bottom deciles of predicted age relative to their chronological age. Interestingly, in memory helper cells and regulatory T cells, we find that donors with longer mean cellular transcript lengths are predicted to have a younger age relative to their chronological age (Figure 6G–6L). This is in agreement with previous reports linking mean cellular transcript length with aging [42, 43].

## DISCUSSION

The immune system ages at many distinct levels including molecular, cellular, compartmental, and systemic [44]. At a molecular level, for example, aging CD8 T cells lose chromatin accessibility at select gene promoters, leading to reduced capacity for oxidative phosphorylation [32, 45]. At a cellular level, T cells progressively accumulate dysfunctional mitochondria [46] and see receptor signaling pathways become dysregulated [47]. At a compartment level, thymic involution leads to a gradual reduction in naïve T cell number and diversity [48]. At a systemic level, senescent cells in the immune system secrete senescence-associated secretory phenotype (SASP) [49] with toxic bystander effects and clonal expansion of unique immune cell clones increases the risk of leukemia [50].

*Tictock* is not intended to function as a global PBMC-level chronological age predictor, but rather as a lineage-restricted, cell-type–specific aging clock that captures relative intrinsic aging states within defined T cell populations. Accordingly, compressed donor-level age ranges and broad single-cell distributions reflect biological constraints and heterogeneity within T cell lineages rather than reduced predictive validity.

Due to this complexity, it is critical to study how aging affects multiple levels of immune organization. It is equally important to study how specific diseases may affect immune aging and the normal functioning of the immune system. Both acute COVID-19 infection [51] and HIV [37] have been reported to accelerate epigenetic aging in immune cells, and antiretroviral therapy may slow it [38]. However, without a precise understanding of how each disease influences specific aspects of immune aging, it is difficult to develop targeted restorative therapies. Deciphering the interplay between different levels of immune aging is also important for a full understanding of age-associated T cell dysfunction.

By developing age prediction models for each of six distinct T cell subsets, we can predict the cell type and age of individual T cells using single-cell transcriptomic data. We opted to automate cell type prediction in addition to age prediction to increase the robustness and reproducibility of our findings. Our results indicate that acute COVID-19 is both a cell-intrinsic and systemic immune aging phenomenon, while long-term HIV infection treated with ART shows an accelerated intrinsic aging signature in naïve CD8 T cells. Although our study evaluates COVID-19 as a unified clinical category, severity-stratified analyses may be informative in larger datasets [12], and we identify this as an avenue for future work. We further find that shorter mean transcript length is a feature of aging lymphocytes and is tracked by our transcriptomic clock.

Because *Tictock* generates six independent age models trained within individual T cell subsets, predicted donor-level ages naturally exhibit a compressed dynamic range relative to chronological age. This reflects limited within-lineage transcriptional variation and the inherent sparsity of single-cell transcriptomes, rather than a loss of biological signal. Accordingly, *Tictock* is optimized for resolving cell-type–specific intrinsic aging and separating intrinsic from compositional effects.

To construct robust age prediction models for distinct T cell subsets, we first filtered genes to retain only those with a correlation with age greater than 0.01 or less than −0.01, yielding 2,947 candidate genes. These genes were then used to train models for six different T cell types. Notably, 209 genes were common across all models, yet each cell type exhibited a unique genetic signature—ranging from 1,124 unique genes in the regulatory T cell model and 2,188 in the memory helper T cell model (Supplementary Figure 6). This gene overlaps not only points to shared molecular mechanisms of aging, but also suggests that cell type-specific processes contribute uniquely to immune aging. This may reflect a core aging signature conserved between T cell subsets, though we are limited in our interpretation due to the sparseness of single-cell transcriptomics data.

It is also somewhat surprising that the T cell type most associated with accelerated aging in both acute COVID-19 and long-term HIV infection is naïve CD8s. Our findings are in accordance with previous literature showing a short-term loss of naïve CD8s in acute COVID-19 [52], but the current knowledge on cell-intrinsic dysfunction of naïve CD8s in either disease context is limited. Lastly, we found that several ribosomal genes that are upregulated during aging are downregulated in the context of HIV + ART. This is in agreement with the fact that several genes downregulated during HIV infection are involved in ribosomal biogenesis [53].

Mechanistically, naïve CD8 T cells appear particularly vulnerable to aging-associated decline due to a convergence of developmental, antigenic, and epigenetic factors. With advancing age, thymic involution sharply reduces thymic output, constricting the naïve CD8 pool and skewing the T-cell compartment toward memory and effector phenotypes [48]. Meanwhile, cumulative antigenic stimulation across life drives compensatory homeostatic proliferation of existing naïve clones, resulting in reduced T-cell receptor diversity and the emergence of senescent, oligoclonal populations [31, 32]. At the molecular level, naïve CD8 T cells exhibit age-related epigenetic remodeling, including altered DNA methylation and histone modifications that restrict chromatin accessibility and transcriptional flexibility [24, 45]. These combined processes erode the capacity of naïve CD8 T cells to preserve a quiescent but responsive state, rendering them especially sensitive to both intrinsic transcriptional aging and systemic inflammatory stressors such as infection or metabolic imbalance.

Though *Tictock* shows significant promise, there are some limitations. Due to the sparse and noisy nature of single-cell RNA sequencing data, predicting age with an accuracy similar to that of epigenetic clocks is challenging. Techniques such as Buckley et al.’s (2023) bootstrapping method reduce transcriptional variation and enhance cell prediction accuracy, though at the cost of losing single-cell resolution. Importantly, *Tictock* was validated using two specific external datasets from COVID-19 and HIV patients.

While the observations reported here do provide unique biological insights, both COVID and HIV represent viral infections and may not capture disease states in a broader context. Accordingly, the current validation scope is limited to viral conditions. Future studies will be needed to test whether *Tictock* generalizes to non-viral contexts such as autoimmune disorders, cancers, and metabolic or inflammatory diseases, which often involve distinct immune remodeling processes. Extending the model to these settings will be essential to establish its robustness and to assess whether intrinsic and systemic immune aging signatures behave similarly across diverse pathological conditions.

Lastly, although we identify and characterize cellular ribosomal signatures of aging using our novel biomarker, we are cognizant of the fact that this family of genes is expressed at a high level and that, given the relatively low sensitivity of single-cell sequencing, we have missed less abundant genes that play a significant role in immune aging.

During preparation of this manuscript, multiple reports were released complementing findings described here. Another group developed a single-cell transcriptomic clock for human PBMCs, but in contrast to our findings, found that COVID infection had a rejuvenative effect on T cells [54]. Another study similarly identified an opposite aging effect based on single-cell vs. bulk transcriptomic readouts [55], reinforcing the importance of understanding the effect of cell type composition on aging measurements.

Beyond its research applications, *Tictock* could be developed into a clinical tool for assessing immune system health and therapy responsiveness. By quantifying both cell-intrinsic and compositional aspects of immune aging, *Tictock* may help enable immune risk stratification in older adults or patients with chronic inflammatory and infectious diseases. Longitudinal tracking of transcriptomic age at the single-cell level could also be used to detect therapy-induced rejuvenation following interventions such as vaccination, hematopoietic stem cell transplantation, or senolytic and metabolic treatments. Ultimately, integrating single-cell transcriptomic clocks into clinical monitoring pipelines may provide a powerful means to evaluate how therapeutic or lifestyle interventions modulate immune aging trajectories in individual patients.

In summary, our study provides a novel framework for understanding immune aging by integrating single-cell transcriptomic data with automated T cell type and age prediction. By highlighting both cell-intrinsic and systemic aspects of immune aging, our approach offers novel insights into how diseases such as COVID-19 and HIV differentially impact the immune system at the cellular level. These findings highlight the potential of single-cell aging biomarkers to improve the specificity of aging diagnostics and to inform therapeutic strategies aimed at mitigating age-related immune decline. Future research should focus on expanding *Tictock* to other cell types and disease conditions to build a more comprehensive map of aging across the immune system, and to explore how therapeutic interventions could potentially reverse or modulate these aging signatures.

## METHODS

### Human cohorts

The Terekhova et al. (2023) dataset was used to train the cell type prediction and age prediction models. This dataset comprised roughly 2 million peripheral blood mononuclear cells from 317 samples from 166 individuals aged 25–85 years old. All participants were healthy Caucasian non-obese non-smokers. Blood was collected between 2018 and 2021 after an overnight fast. The Chromium Single Cell 5’ v2 Reagent Kit from 10x Genomics was used to generate single-cell transcripts. The Yasumizu et al. (2024) dataset was used to validate the accuracy of our cell type prediction and age prediction models and contained 1.8 million CD4+ T cells generated using the Chromium Next GEM Single Cell 5′ Kit v2.

The Ren et al. (2021) dataset made up of 284 samples from 171 COVID-19 patients and 25 healthy individuals served as an external validation dataset for our model. The samples from COVID-19 patients were categorized into moderate convalescence (*n* = 89), moderate progression (*n* = 33), severe convalescence (*n* = 51) and severe progression (*n* = 83) according to severity and stage. They then generated a transcriptomic dataset of 1.46 million immune cells using Chromium Single Cell 3’ v2 Reagent, Chromium Single Cell 3’ v3 Reagent, Chromium Single Cell 5’ v2 Reagent, and Chromium Single Cell V(D)J Reagent kits from 10x Genomics. For our analysis, we included only cells derived from patients who had COVID-19 within 60 days of sample collection. This window was chosen to focus on acute and earlier convalescent immune responses, while excluding later time points in which long-term immune remodeling, recovery heterogeneity, and treatment effects introduce substantial confounding. Furthermore, we limited our analysis to cells processed with the Chromium Single Cell 5’ V2 kit.

The Wang et al. (2024) dataset included fourteen donors, eight of whom had HIV. Of the eight individuals with HIV, seven are actively treated with ART. The dataset contained 262,818 PBMCs from these donors generated using the Chromium 5′ Single Cell Gene Expression system from 10X Genomics.

### Data processing

All datasets were filtered to remove cells with high mitochondrial reads and low transcript counts (<2000 reads). The original Terekhova et al. (2023) preprocessing pipeline also removed doublets and multiplets based on total UMI distributions and Scrublet-derived scores; therefore, we did not apply an additional doublet-calling step in our downstream analyses. Datasets were then processed using the Seurat [56] pipeline, which included NormalizeData, ScaleData, FindVariableFeatures, RunPCA, FindNeighbors, FindClusters, and RunUMAP functions to identify cell types. For the Terekhova et al. (2023) dataset, donor-level batch correction was performed using Harmony-based integration in Seurat prior to training/testing split, ensuring that cell-type clustering was not driven by batch effects. For datasets including other peripheral blood mononuclear cell subpopulations, only T cells were retained. The original Terekhova et al. (2023) dataset used for model training was filtered to 2,947 genes that showed at least a weak correlation with age (|R| >0.01). This filtering was applied to minimize technical noise and ensure consistent input features for both cell-type and age-prediction models. Canonical lineage-defining markers were retained above this threshold.

### Cell type annotation

Cell type annotation was conducted using the ‘Seurat’ package (v5.1.0) in R. Gene expression matrices were loaded and aligned with the metadata. T cell subsets were re-annotated for more specificity and clustered into six T cell subsets. Cell clustering was performed with the ‘FindNeighbors’ and ‘FindClusters’ functions from Seurat (34) followed by visualization using UMAP. Clusters were manually annotated and renamed based on marker gene (CD8A, CD4, CCR7, GZMB, GNLY, FOXP3) expression. The re-labeled T cell subsets were incorporated into the metadata and used for training the cell type prediction model.

### Cell type and age prediction models

The dataset was split into training and test sets based on donor ID, with 80% of the donor IDs assigned to the training dataset and 20% assigned to the test dataset. For cell type prediction, we trained a multinomial logistic regression model using the ‘glmnet’ package in R (cv.glmnet, family = “multinomial”). The model was fit on the full set of 2,947 age-correlated genes (|R| >0.01) plus a binary sex covariate, with all predictors internally standardized by glmnet and λ selected by cross-validation.

For age prediction, we built six independent models—one for each T cell subset (naïve CD4, memory CD4, naïve CD8, effector CD8, memory CD8, and regulatory T cells)—using the Terekhova et al. (2023) data. For each subset, we used ‘cv.glmnet’ with an elastic net penalty (α = 0.5) to predict donor age from the expression of the same 2,947 genes. From this initial model, we extracted all non-zero coefficients and then refitted a second ‘cv.glmnet’ model using only these selected features. The resulting refit models were saved and applied to all external datasets for age prediction [57].

To control for differences in donor chronological age and cell type composition when comparing disease conditions, we computed age residuals by regressing predicted transcriptomic age on chronological age and predicted cell type. All disease-associated aging analyses in the manuscript use these residuals, which represent cell-intrinsic transcriptional aging independent of donor age. Accuracy was defined using the mean absolute error (MAE), with MAE ≤12 years considered strong, 12–15 years moderate, and >15 years weak. For correlation, we classify R ≥0.70 as strong, 0.40–0.70 as moderate, and <0.40 as weak.

### Enrichment analyses

Gene ontology (GO) term enrichment analysis was performed on shared genes using the ‘enrichGO’ function from the ‘clusterProfiler’ package in R [58]. A chromosome visualization plot was created using the ‘karyoploteR’ package in R where the gene locations were plotted on a karyoplot [59]. Genes included were those used as coefficients across all six T cell models.

### Transcript length estimation

Gene-level transcript lengths were obtained from Ensembl (GRCh38) using the R package biomaRt. For each gene, we defined a representative transcript length as the maximum annotated length across all isoforms. For each cell, raw counts were scaled to a fixed library size (10,000 counts per cell; CPM-like normalization), and the expression-weighted mean transcript length was calculated as the column-sum of (scaled expression × gene length). These per-cell means were then summarized per donor and cell type for downstream analyses.

### Statistics

To determine the statistical significance of the differences in residuals between conditions, pairwise *t*-tests were performed with Bonferroni correction to adjust for number of cell types analyzed. ANCOVA tests were conducted to measure the differences in cell proportions while accounting for the confounding effect of age by adjusting for age-related variability. The ‘aov’ function was used from the ‘stats’ package in R. Additionally, the ‘emmeans’ package provided adjusted mean estimates and *p*-values to compare between conditions [60]. The ‘ggplot2’ package was utilized for graphing.

For gene expression, residual, and cell proportion analyses, cells were sampled to maintain the same number of cells per donor. ANCOVA was used to compare proportions between conditions (Control vs. HIV) while adjusting for age. Statistical analysis was performed using ‘emmeans’ [60] for pairwise comparisons and car for variance inflation factor (VIF) calculations. Partial correlations were calculated to control for confounding factors (donor age or condition) for gene expression analysis using the ‘ppcor’ package.

### Data availability

The Terekhova et al. (2023) data were available through the Synapse platform (syn49637038). The Ren et al. (2021) data were obtained on the Gene Expression Omnibus (GSE158055). The Wang et al. (2024) dataset was accessed from the Gene Expression Omnibus (GSE243905).

### Code availability

The code used to build the model and perform all analyses will be publicly available on GitHub prior to publication. The primary programming languages used were R and Python with freely available software packages. All computational work was performed on Amazon Web Services (AWS) Elastic Compute Cloud (EC2) instances.

## Supplementary Materials

Supplementary Figures
