# Supplementary material for: Single-cell transcriptomics reveal intrinsic and systemic T cell aging in COVID-19 and HIV
Source: Aging (Albany NY). 2026 Feb 8;18(1):5–28. doi: 10.18632/aging.206353 (PMC13285955; doi:10.18632/aging.206353)
Supplement: Supplementary Figures [file aging-18-1-206353-s001.pdf]

SUPPLEMENTARY FIGURES

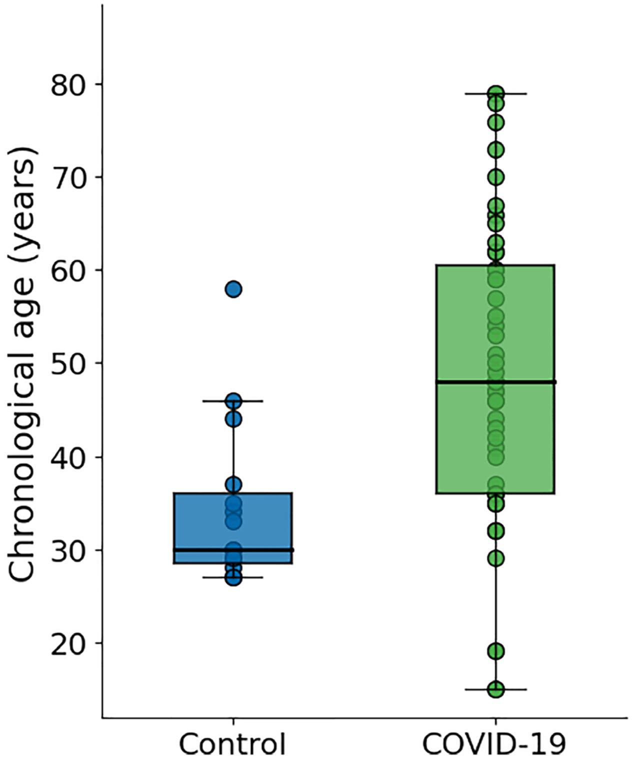

Supplementary Figure 1. Chronological age distributions of COVID-19 and control donors. A donor-level comparison of chronological ages for patients with COVID-19 and healthy controls from the Ren et al. dataset after applying quality filters. Each point represents one donor, with boxplots summarizing the age distribution within each group. COVID-19 donors were significantly older than controls ($p = 6.44 \times 10^{-5}$ ***), confirming that chronological age differs between groups.

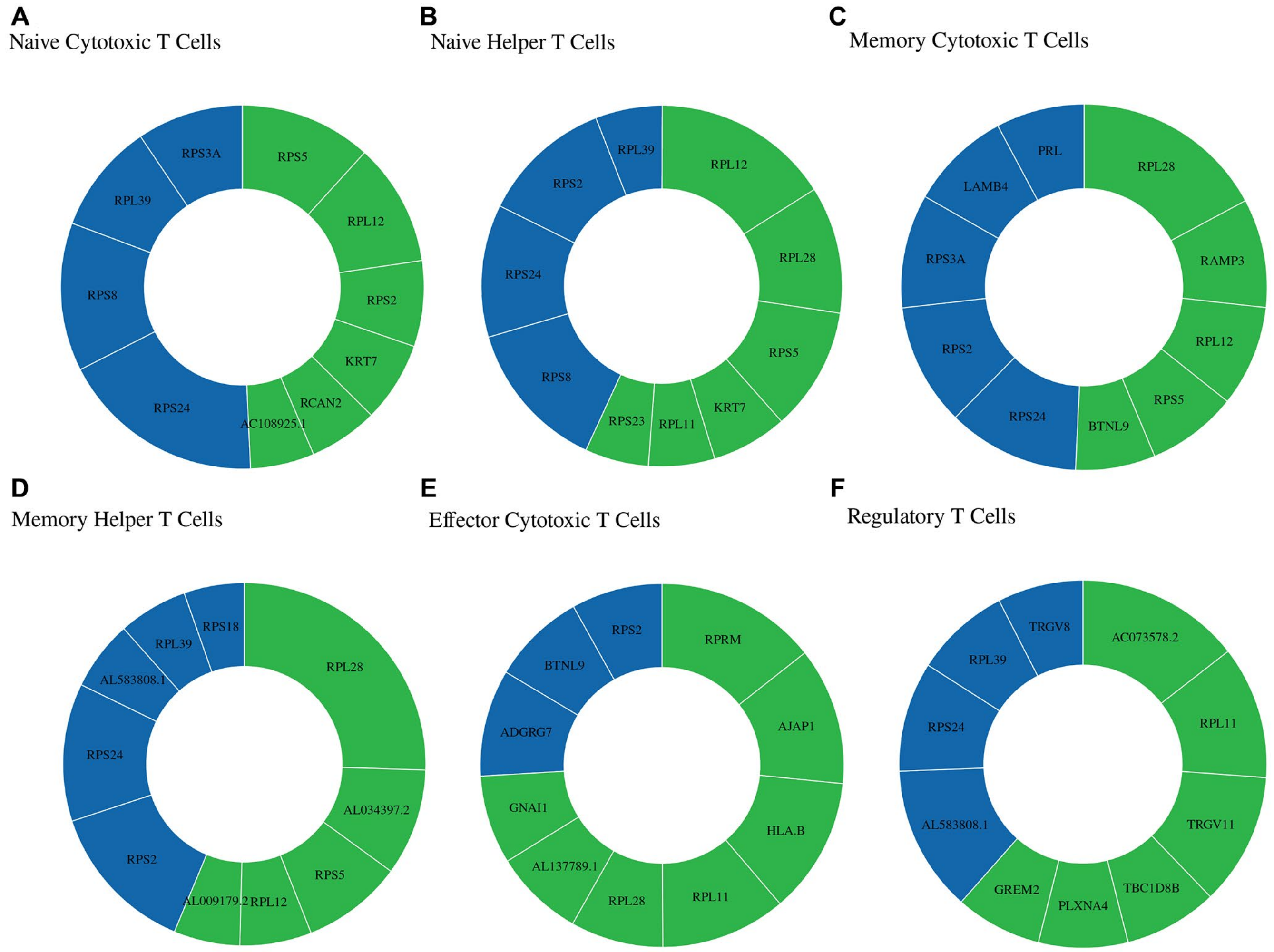

Supplementary Figure 2. Gene contributions across T cell type-specific age prediction models. (**A**–**F**) Donut plots illustrating the top ten most influential gene coefficients for each T cell type–specific age prediction model: (**A**) naïve cytotoxic, (**B**) naïve helper, (**C**) memory cytotoxic, (**D**) memory helper, (**E**) effector cytotoxic, and (**F**) regulatory. Upregulated genes are shown in green and downregulated genes in blue. Gene size reflects the relative magnitude of the model coefficient, highlighting the primary drivers of age prediction within each T cell subset.

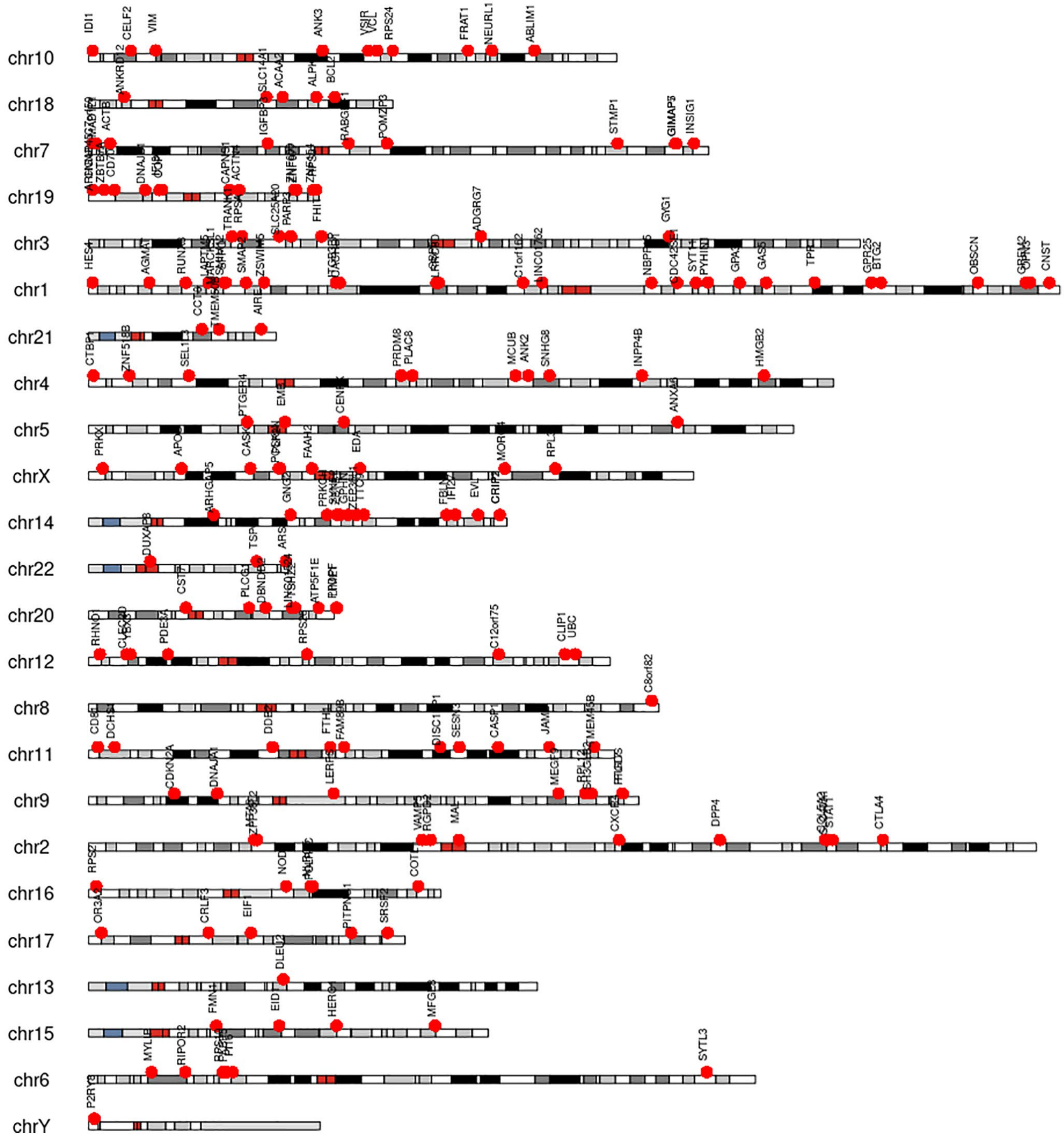

Supplementary Figure 3. Predictive genes localized to regions of the genome. Karyoplot mapping chromosomal locations of shared predictive genes identifies dense regions on chromosomes 1 and 14.

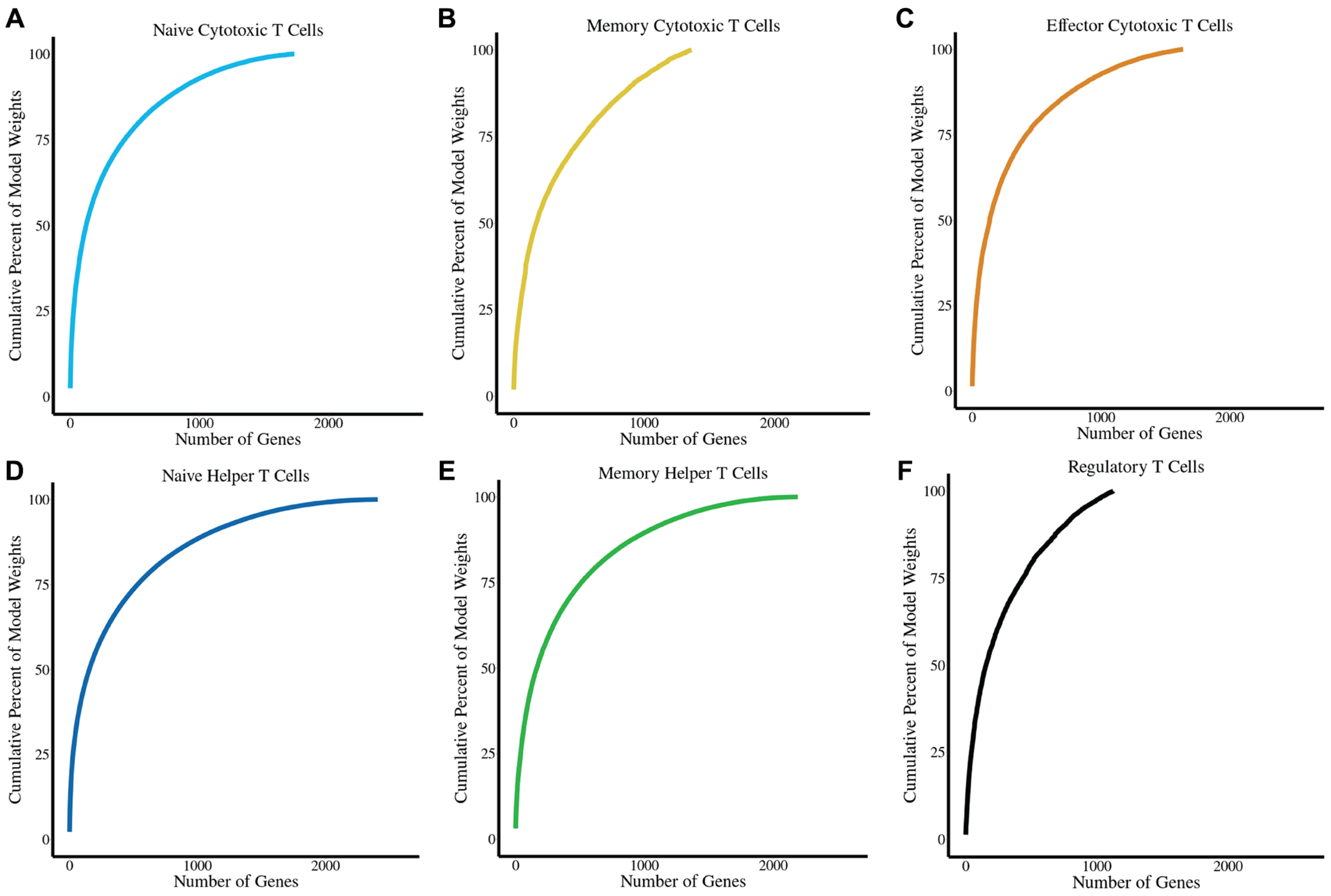

Supplementary Figure 4. Distribution of model weights across genes. (**A**–**F**) Cumulative distribution functions showing how age prediction model weights are distributed across all genes for each T cell subset: (**A**) naïve cytotoxic, (**B**) memory cytotoxic, (**C**) effector cytotoxic, (**D**) naïve helper, (**E**) memory helper, and (**F**) regulatory. Each curve represents the cumulative proportion of total model weight explained as genes are ranked by absolute coefficient magnitude, illustrating differences in weight concentration across T cell–specific aging models.

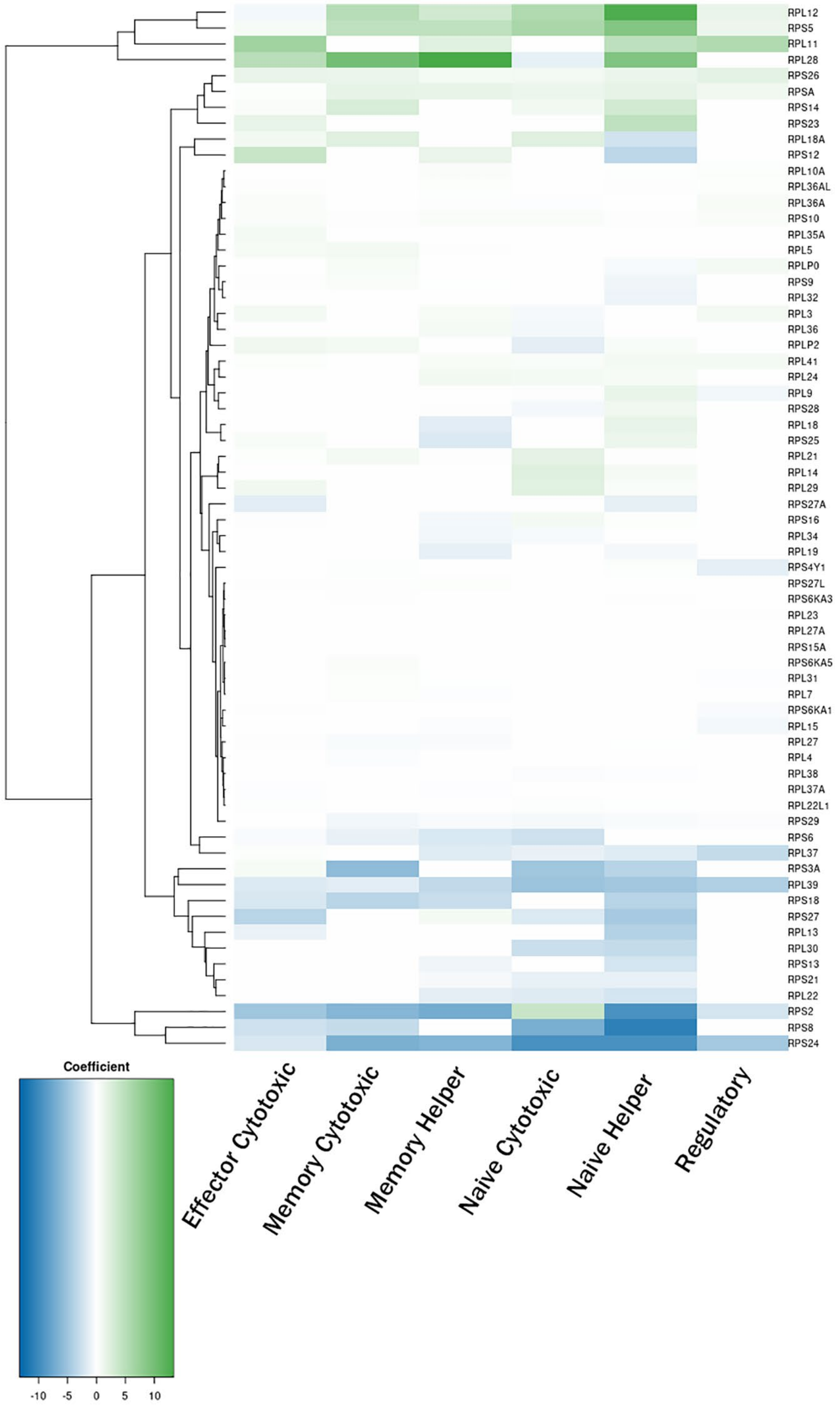

Supplementary Figure 5. Ribosomal gene coefficients across T cell subsets. Heatmap depicting the age prediction coefficients assigned to ribosomal genes for each T cell subset in the Terekhova et al. (2023) dataset. The heatmap reveals patterns of ribosomal gene contributions to age prediction across different T cell types.

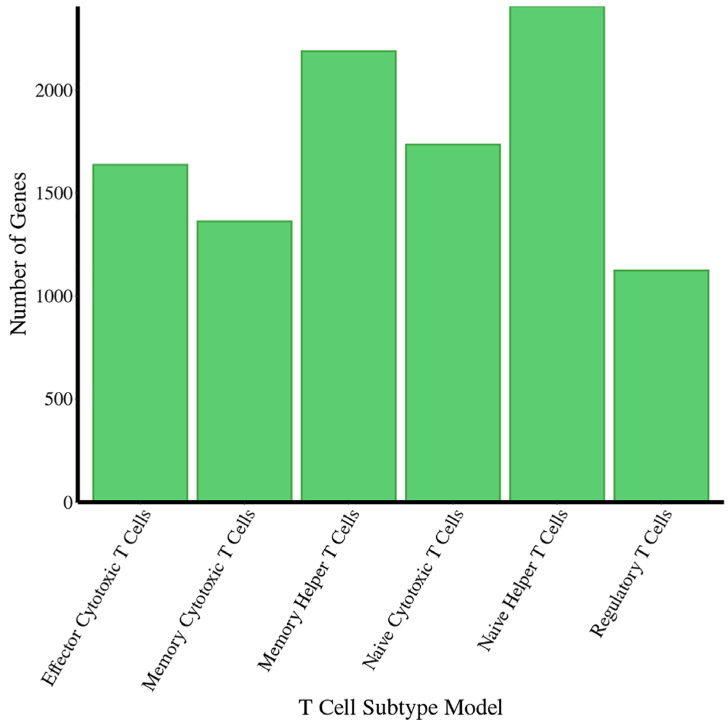

Supplementary Figure 6. Quantified genes for T cell age prediction models. The number of genes unique to each of the six T cell subset age prediction models after training.
